# Emerging roles of alternative RNA splicing in oral squamous cell carcinoma

**DOI:** 10.3389/fonc.2022.1019750

**Published:** 2022-11-25

**Authors:** Miaomiao Liu, Jihua Guo, Rong Jia

**Affiliations:** ^1^ The State Key Laboratory Breeding Base of Basic Science of Stomatology (Hubei-MOST) & Key Laboratory of Oral Biomedicine Ministry of Education, School & Hospital of Stomatology, Wuhan University, Wuhan, China; ^2^ Department of Endodontics, School & Hospital of Stomatology, Wuhan University, Wuhan, China; ^3^ RNA Institute, Wuhan University, Wuhan, China

**Keywords:** alternative splicing, OSCC, splicing factor, tumor progression, therapeutic targets

## Abstract

Alternative RNA splicing (ARS) is an essential and tightly regulated cellular process of post-transcriptional regulation of pre-mRNA. It produces multiple isoforms and may encode proteins with different or even opposite functions. The dysregulated ARS of pre-mRNA contributes to the development of many cancer types, including oral squamous cell carcinoma (OSCC), and may serve as a biomarker for the diagnosis and prognosis of OSCC and an attractive therapeutic target. ARS is mainly regulated by splicing factors, whose expression is also often dysregulated in OSCC and involved in tumorigenesis. This review focuses on the expression and roles of splicing factors in OSCC, the alternative RNA splicing events associated with OSCC, and recent advances in therapeutic approaches that target ARS.

## 1 Introduction

**Figure 1:**
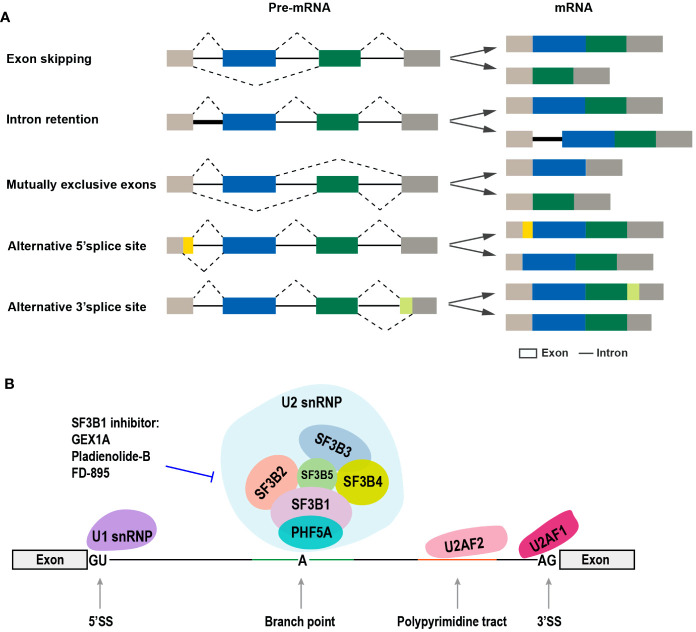
The common types of alternative RNA splicing and the SF3b complex involved in alternative RNA splicing. **(A)** The different types of alternative RNA splicing and transcript variants (exon skipping, intron retention, mutually exclusive exons, alternative 5’ splice site, and alternative 3’ splice site). **(B)** Diagram of the components of spliceosome complex, especially SF3b complex, and SF3b1-specific inhibitors.

Oral squamous cell carcinoma (OSCC) is among the most malignant cancer types worldwide (1), and its prognosis remains poor despite improvements in therapies in recent decades. Therefore, the molecular pathogenesis of the development of OSCC needs to be urgently determined. The dysregulated alternative RNA splicing (ARS) of pre-mRNA contributes to the development of many cancer types, including OSCC (2, 3). The alternative splicing of pre-mRNA in eukaryotes was first discovered in 1980 (4, 5), by which exons can be jointed in different ways to produce multiple mRNA isoforms, and then possibly encode different protein isoforms (6). As a complex biological process, ARS is tightly regulated *via* interactions between *cis*-elements and *trans*-acting splicing factors. The *cis*-elements include exon splice enhancer, exon splice silencer, intron splice enhancer, and intron splice silencer, while *trans*-elements include *trans*-acting splicing factors (7). The common types of alternative RNA splicing include exon skipping, mutually exclusive exons, alternative 5’ splice site, alternative 3’ splice site, and intron retention (**Figure 1A**). ARS occurs in more than 90% human genes and is precisely regulated in cells. This review focuses on the expression and roles of splicing factors in OSCC, the ARS events associated with OSCC, and recent advances in therapeutic approaches that target ARS.

## 2 Dysregulation of ARS in OSCC progression

The ARS patterns of pre-mRNA are often altered in OSCC, mainly because of the dysregulated expression of splicing factors. Moreover, abnormal alternative RNA splicing can promote the development of OSCC by enhancing cancer cell proliferation, invasion, and metastasis.

### 2.1 Spliceosome complex

The process of pre-mRNA alternative splicing is catalyzed by the spliceosomes, which are divided into two categories, namely, major spliceosomes (U1, U2, U4, U5, and U6 small nuclear ribonucleoproteins, snRNPs) and minor spliceosomes (U11, U12, U4atac, and U6atac snRNPs) (8) (**Figure 1B**). Spliceosome is a megadalton complex. The SF3b protein subcomplex is a component of the U2 snRNP and plays key roles in recognizing the branch point sequence during spliceosome assembly (9). SF3B1 is the largest protein in the SF3b complex and has been regarded as a therapeutic target because of its frequent mutations in both hematologic cancers and solid tumors (10–13). The high expression level of SF3B1 in hepatocellular carcinoma (HCC) has been correlated with increased tumor aggressiveness and shorter overall survival (OS) (14). SF3B1 is also upregulated in OSCC tissues (15).

### 2.2 Splicing factors

The ARS of pre-mRNA is mainly regulated by splicing factors. Splicing factors include serine/arginine-rich protein (SR) family and heterogeneous nuclear ribonucleoproteins (hnRNPs) family, and other splicing factors.

#### 2.2.1 SR family

SR proteins are an evolutionarily conserved family and consist of at least 12 members (SRSF1 to SRSF12). SR proteins contain N-terminal RNA recognition motifs and C-terminal serine and arginine-rich (RS) domains. SR proteins play important roles in the regulation of pre-mRNA splicing and ARS (16). Several SR family members participate in the tumorigenesis of OSCC.

##### 2.2.1.1 SRSF1

SRSF1 is a well-known oncogenic splicing factor (17). SRSF1 is also overexpressed in OSCC and promotes cell proliferation, invasion, and epithelial-mesenchymal transition (EMT) by interacting with lncRNA LINC01296 (18).

##### 2.2.1.2 SRSF3

Oncogene SRSF3 is overexpressed in many cancers, including OSCC (19–21). In OSCC, SRSF3 overexpression is positively associated with high-grade tumors and lymph node metastasis, indicating that SRSF3 may promote the development of OSCC. Mechanically, SRSF3 is required for the expression of Slug and N-cadherin genes (19), which can promote EMT and metastasis. Moreover, SRSF3 can inhibit autophagy by repressing the expression of p65 and FOXO1, and their downstream target, BECN1, which is a key factor of autophagy (22). In general, autophagy plays a tumor-suppressive function in the early stage of oncogenesis (23). SRSF3 may enhance the early development of OSCC by inhibiting autophagy. Importantly, SRSF3 has an autoregulatory mechanism to maintain its stable expression level in cells. SRSF3 exon 4 is an alternative exon with an in-frame pre-mature stop codon. SRSF3 inhibits its own expression by increasing exon 4 inclusion. In cancer cells, oncogenic PTBP1 and PTBP2 proteins impair SRSF3 autoregulation by repressing SRSF3 exon 4 inclusion and significantly enhancing SRSF3 expression (21).

##### 2.2.1.3 SRSF5

SRSF5 is also overexpressed in OSCC and is essential for cell proliferation and tumor development. The downregulation of SRSF5 expression significantly inhibited cell growth and cell cycle progression, and tumor formation *in vivo*. Interestingly, SRSF5 also has an autoregulatory mechanism mediated by the selection of two alternative 3’ splice sites in exon 6. The usage of proximal 3’ splice site includes a pre-mature stop codon. SRSF3 inhibits the usage of proximal 3’ splice site and the autoregulation, thus remarkably enhancing SRSF5 expression in cancer cells (24).

#### 2.2.2 Heterogeneous nuclear ribonucleoprotein family

Heterogeneous nuclear ribonucleoproteins (hnRNPs) represent a large family with multiple functions in regulating RNA metabolism, including ARS. The proteins in this family are named from A1 to U (25). Many hnRNP proteins are associated with tumorigenesis, and some of them have been demonstrated to be oncogenes (26). Some hnRNP proteins play oncogenic roles, while others play tumor-suppressive roles in OSCC.

##### 2.2.2.1 HnRNP A1

HnRNP A1 is overexpressed in OSCC tissues and required for OSCC cell proliferation. HnRNP A1 is predominantly highly expressed in the G2/M cell cycle phase. A1 knockdown leads to cell cycle G2/M arrest in OSCC cells. In principle, A1 promotes alternative exon 5 inclusion of cyclin-dependent kinase 2 (CDK2) and produces full-length CDK2 protein, which participates in cell cycle regulation (27).

##### 2.2.2.2 HnRNP C

HnRNP C is also overexpressed in OSCC tissues. The high expression of hnRNP C is associated with advanced tumor stage, lymph node metastasis, and poor prognosis in patients with OSCC. HnRNP C promotes OSCC cell proliferation, migration, and invasion (28). In addition, hnRNP C can enhance the radioresistance of OSCC cells by interacting with lncRNA LINC00662 to stabilize adenylate kinase 4 (AK4) mRNA and increase the expression of AK4 protein, which is a key enzyme in cancer cell resistance to radiation (29).

##### 2.2.2.3 HnRNP D

The nuclear expression of hnRNP D is remarkably higher in oral dysplasia and OSCC tissues than in normal tissues and is positively correlated with OSCC tumor size, tumor stage, and poor patient survival (30). The overexpression of hnRNP D is mediated by NF-κB (RelA) binding to its promoter. HnRNP D expression is strongly correlated with NF-κB in OSCC (31).

##### 2.2.2.4 HnRNP K

HnRNP K mainly plays oncogenic roles in cancer (32). In comparison with normal oral mucosal tissues, hnRNP K is overexpressed in OSCC tissues (33). HnRNP K protein can be detected in both cytoplasm and nucleus. Both cytoplasm and nucleus hnRNP K expression gradually increases from normal tissue to OSCC, and is associated with poor OSCC prognosis (33, 34).

##### 2.2.2.5 HnRNP L

HnRNP L is overexpressed in OSCC tissues and required for OSCC cell growth, migration, and tumorigenesis (35). HnRNP L expression is also controlled by an autoregulatory mechanism. HnRNP L promotes the inclusion of its alternative exon 7, which includes a pre-mature stop codon. Only the transcripts without exon 7 can encode the full-length oncogenic hnRNP L protein. In OSCC cells, increased SRSF3 promotes exon 7 skipping and the expression of full-length hnRNP L protein, and then enhances tumorigenesis (36).

##### 2.2.2.6 HnRNP E1

Most splicing factors are overexpressed and play oncogenic roles in OSCC. However, hnRNP E1, which is also called PCBP1, mainly functions as a tumor suppressor (37). HnRNP E1 overexpression significantly inhibited OSCC cell proliferation and tumor formation by inhibiting the usage of the proximal 5’ splice site of STAT3 exon 23 and the expression of oncogenic STAT3α isoform (38). Importantly, two leucine residues (Leu100 and Leu102) of PCBP1 protein are frequently mutated in cancers and are essential for PCBP1 function (39).

##### 2.2.2.7 HnRNP G

HnRNP G, which is also known as RBMX, is differentially expressed in various cancer types. For example, hepatocellular carcinoma has a high level of hnRNP G (40), whereas bladder cancer shows downregulated hnRNP G expression (41). In dysplastic or OSCC tissues, the expression levels of hnRNP G protein are remarkably lower than those in normal oral mucosal tissues. The ectopic expression of hnRNP G remarkably abolished the clonogenic capacity of OSCC cells in soft agar *in vitro*, suggesting that hnRNP G may be a tumor suppressor in OSCC (42). Interestingly, in the patients of TCGA head and neck squamous cell carcinoma (HNSCC), the lower transcriptional level of hnRNP G is significantly associated with favorable overall survival (43). Therefore, further study is required for the complete understanding of the expression and clinical effects of hnRNP G in OSCC.

#### 2.2.3 Other splicing factors

In addition to SR and hnRNP protein families, some other splicing factors are also associated with OSCC.

SF3B1 is a component of SF3b complex and is involved in the recognition of branch point during spliceosome assembly. SF3B1 expression is significantly upregulated in OSCC tissues compared with normal adjacent tissues (15). Interestingly, SF3B1 is also identified in the cultured medium based on a secretome analysis of HNSCC cell lines (15). Moreover, SF3B3, another component of SF3b complex, is also significantly upregulated in OSCC (15) and may be mutated in some cases (44). Further study is needed for the complete understanding of roles of SF3B1 and SF3B3 in OSCC.

LSM12 is a member of Sm-like proteins and regulates pre-mRNA splicing. LSM12 is significantly upregulated in OSCC tissues of patients and animal models. LSM12 is required for OSCC cell proliferation, migration, and tumorigenesis (45).

Epithelial splicing regulatory protein 1 and 2 (ESRP1 and ESRP2), which are also known as RBM35A and RBM35B, were upregulated in OSCC compared with normal epithelium. However, they were downregulated in the invasion front. ESRP1 and ESRP2 do not affect cell proliferation but inhibit cancer cell migration (46). Mechanically, ESRP1 knockdown significantly increased RAC1 exon 3b inclusion and the expression level of RAC1b isoform, which can modulate actin and enhance cell migration (46).

Therefore, splicing factors play significant roles in OSCC progression. However, the underlying mechanisms in which splicing factors are mediated need to be further investigated. **Table S1** summarizes the information related to splicing factors in OSCC.

### 2.3 ARS events associated with OSCC

**Figure 2:**
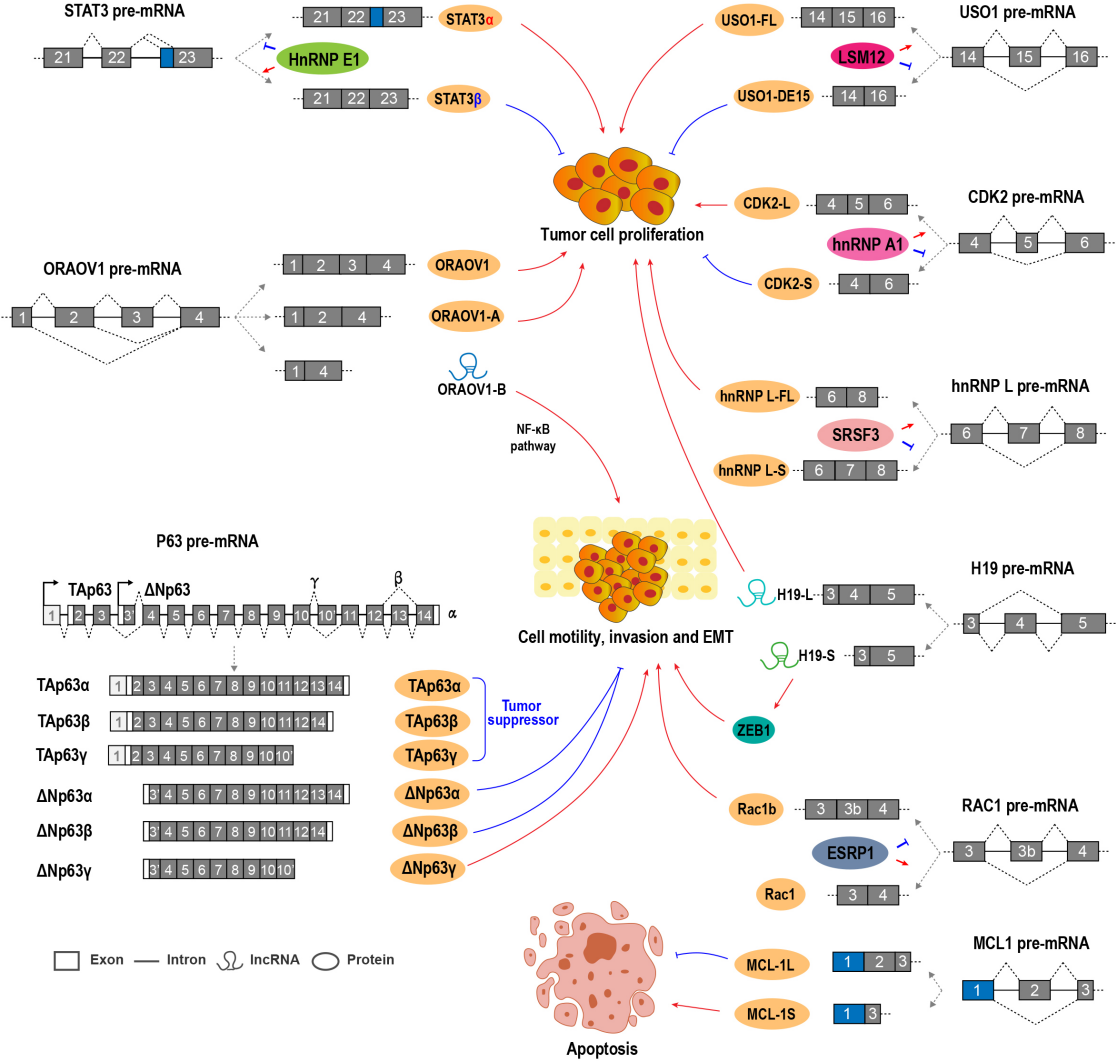
Alternative RNA splicing and splicing factors regulate OSCC cell proliferation, invasion, and EMT.

In addition to splicing factors, many alternative RNA splicing events of cancer-related genes contribute to OSCC progression (**Figure 2**; **Table S2**).

#### 2.3.1 Alternative spliced events of cancer-related genes associated with OSCC proliferation and apoptosis

##### 2.3.1.1 MCL1

Myeloid cell leukemia-1 (MCL1) belongs to the Bcl-2 family and is an anti-apoptotic regulator essential for cell proliferation. MCL1 has three splicing variants, including MCL-1L, MCL-1S, and MCL-1ES (47). MCL-1L contains all exons, whereas MCL-1S is a short isoform without exon 2 and encodes a pro-apoptotic protein. MCL-1ES is another short isoform with a truncated exon 1 and encodes a pro-apoptotic protein, which is expressed at a very low level in OSCC (48). OSCC tissues expressed significantly higher MCL-1L level than normal adjacent control tissues (49). The downregulation of MCL-1L could enhance the radio sensitivity of OSCC cells (48). High MCL-1L expression is positively associated with lymph node positivity, tumor size, and poor overall survival in patients with OSCC. MCL-1L is an independent prognostic factor for OSCC (50). Conversely, MCL-1S expression is much weaker than MCL-1L in OSCC cells and tissues. The ratio of MCL-1L/S was positively correlated with poor overall survival (50). Mechanically, the MCL-1S protein can dimerize with MCL-1L and neutralize its anti-apoptotic function (51).

##### 2.3.1.2 Survivin

Survivin is an anti-apoptosis protein and is encoded by BIRC5 (baculoviral inhibitor of apoptosis protein repeat-containing 5) gene. Six splicing variants have been reported, including survivin-wt, survivin-2B, survivin-ΔEx3, survivin-3B, survivin-2α, and survivin-3α. Survivin-wt is a conventional variant composed of exons 1 to 4. Survivin-2B contains part of the intron 2 sequence and encodes a bigger protein with markedly reduced anti-apoptosis capacity. Survivin-ΔEx3 is a short isoform caused by exon 3 skipping, but it shows anti-apoptosis capacity (52). Survivin-3B (53) and survivin-2α (54) comprise extra sequences from intron 2 and intron 3, respectively. Survivin-3B is anti-apoptotic, while survivin-2α is pro-apoptotic. In OSCC cells and tissues, survivin-wt, survivin-ΔEx3, and survivin-2B are the dominantly overexpressed isoforms (55, 56). The expression level of survivin-3B in OSCC is positively associated with poor differentiation and lymph node metastases (55, 56). However, pro-apoptotic survivin-2α and survivin-3α were also highly expressed in OSCC tissues (55). The roles and regulatory mechanisms of survivin isoforms in OSCC remain to be further explored.

##### 2.3.1.3 STAT3

Transcription factor STAT3 plays important roles in the initiation and development of tumors. STAT3 has two isoforms generated by the ARS of exon 23. STAT3α is the longer isoform and encodes the full-length oncogenic STAT3α protein. STAT3β is shorter and encodes the truncated and tumor-suppressive STAT3β protein. Splicing factor hnRNP E1 (PCBP1) can bind to an exonic splicing suppressor (ESS) in STAT3 exon 23 and repress the expression of STAT3α in OSCC cells (38, 39).

##### 2.3.1.4 USO1

USO1 (vesicle transport factor), which is also known as P115/TAP, is a member of the tether protein family, and it plays oncogenic roles in multiple myeloma and colon cancer (57, 58). USO1 exon 15 is an alternative exon. Full-length USO1 isoform was positively associated with OSCC cell proliferation and enhanced tumorigenic capacity, while USO1 short isoform without exon 15 showed the opposite effects on OSCC cell proliferation and cell cycle progression. LSM12 promotes USO1 exon 15 inclusion (45).

##### 2.3.1.5 H19

H19 is an lncRNA that can function as an oncogene in OSCC and is required for OSCC cell proliferation, invasion, and migration (59). Interestingly, H19 exon 4 is an alternative exon. H19-S, a short transcript isoform of H19 lncRNA that lacks exon 4, was able to transform normal oral mucosal cells and promote OSCC cell growth. H19-S could also induce EMT and promote oral carcinogenesis by binding and stabilizing zinc-finger E-box binding homeobox 1 (ZEB1) mRNA and increasing ZEB1 expression compared with the full-length transcript H19-L (60).

#### 2.3.2 Alternative spliced events of cancer-related genes associated with OSCC invasion and metastasis

##### 2.3.2.1 P63

P63 is a tumor suppressor gene and bears strong homology to p53 (61). However, the exact function of p63 in OSCC is quite complicated because of the multiple isoforms of p63 (62). Two promoters of p63 drive the expression of two types of isoforms, namely, TAp63 and ΔNp63 (63, 64). Each of these isoforms has at least three variants (α/β/γ), which encode proteins with different COOH-termini generated by ARS. TAp63 isoforms are generally tumor suppressors. ΔNp63α is the predominant splicing isoform expressed in OSCC cells and tissues. In OSCC cells, Snail repressed ΔNp63α expression by inhibiting the binding of C/EBP to the promoter of ΔNp63α, and then increased cellular epithelial-mesenchymal transition and invasive capacity (65). ΔNp63β overexpression reversed stromal cell-like to epithelial cells in OSCC (66). The stable overexpression of ΔNp63β inhibited OSCC cell invasion and metastasis through the downregulation of Wnt5A, Ror2, and MMP-2 (66, 67). ΔNp63β can upregulate miR-205, which downregulates zinc-finger E-box binding homeobox (ZEB) 1 and ZEB2 and then inhibits EMT (68). However, ΔNp63γ promotes EMT (69). Two additional isoforms without exon 4, Δ4Tap63 and ΔNp73L, were also reported. These two isoforms are more likely to occur in patients with metastases (63), but their function and regulatory mechanisms remain unclear.

##### 2.3.2.2 Oral cancer overexpressed 1

Oral cancer overexpressed 1 (ORAOV1) is an oncogene that is required for OSCC cell proliferation, tumor growth, and tumor angiogenesis (70). ORAOV1-A, a splice variant of ORAOV1 without exon 3, encodes a truncated protein because of a pre-mature stop codon in exon 4. ORAOV1-A expression is associated with poor differentiation in OSCC (71). ORAOV1-B, another splice variant of ORAOV1 without exon 2 and exon 3, was validated as an lncRNA. ORAOV1-B is overexpressed in OSCC and positively related with invasion and metastasis. ORAOV1-B can enhance EMT by activating the NF-κB pathway (72). All ORAOV1 isoforms play positive roles in OSCC progression.

## 3 Targeted therapy of OSCC based on ARS

Some antisense oligonucleotides (ASOs) and small molecules have been developed to control alternative RNA splicing in OSCC. ASOs are usually used to target specific sequences in mRNA, whereas small molecules often target splicing factor proteins.

### 3.1 Antisense oligonucleotides

ASOs have been used for the treatment of diseases caused by abnormal alternative RNA splicing, such as spinal muscular atrophy (SMA) (73) and Duchenne muscular dystrophy (DMD) (74). ASOs can bind to the key regulatory elements of target RNA and control ARS. In our previous study, an ASO that targeted the exonic splicing suppressor (ESS) motif of splicing factor SRSF3 pre-mRNA promoted the SRSF3 exon 4 inclusion and reduced the expression level of full-length SRSR3 (75). OSCC cells treated with anti-SRSF3 ASO grew significantly slower compared with those treated with non-specific ASO. Furthermore, anti-SRSF3 ASO treatment significantly sensitized OSCC cells to the chemotherapy drug paclitaxel, thus improving the therapeutic effects of paclitaxel (76).

### 3.2 Small molecules

Small molecules are usually used to inhibit splicing factors. For example, a novel SRSF3 inhibitor SFI003 was developed by screening compounds based on the structure of SRSF3 protein and chemical optimization. SFI003 binds to SRSF3 protein and leads to its degradation. SFI003 induced apoptosis and exhibited potent antitumor efficacy in colorectal cancer cells (77).

The SF3b spliceosome complex is involved in RNA splice site selection, and SF3B1 is a core component of SF3b complex. Pladienolide B, FD-895, GEX1A, and sudemycin E (an analog of FR901464) are inhibitors of SF3B1, and these molecules interact with SF3B1 demonstrating significant induction of cell apoptosis in cancers (78–80). Besides, small-molecule inhibitors of SF3B1 altered MCL1 splicing, thus promoting the generation of the pro-apoptotic variant MCL1-S and diminishing the anti-apoptotic variant MCL1-L, such as spliceostatin A (a methyl ketal derivative of FR901464) in chronic lymphocytic leukemia cells (81), meayamycin B (an analog of FR901464) in non-small cell lung cancer cells (82), and head and neck cancer cells (83). The splice modulators used in different types of tumor cells are summarized in **Table S3**.

## 4 Conclusion and prospects

In conclusion, the dysregulation of ARS occurs universally in OSCC, and may serve as biological markers for the diagnosis and prognosis of OSCC. Oncogenic isoforms produced by ARS can promote the progression of OSCC. Therefore, correcting aberrant alternative RNA splicing and decreasing the expression levels of oncogenic isoforms may contribute to OSCC treatment. Targeting the overexpressed oncogenic splicing factors may also provide novel treatment opportunities in OSCC. However, the regulatory mechanisms of aberrant ARS in OSCC remain largely unclear, thus limiting the development of efficient treatment methods. The regulatory elements of ARS events and corresponding splicing factors need to be explored. Recently, neoantigens produced by ARS have attracted great attention in cancer immunotherapy (84). The modification of RNA splicing can produce *bona fide* neoantigens and induce anti-cancer immunity (85), which sheds light on immunotherapy for OSCC.

## Author contributions

ML wrote the manuscript. JG and RJ edited the manuscript. All authors contributed to the article and approved the submitted version.

## Funding

This work was supported by the Key Research and Development Program of Hubei Province (grant no. 2020BCB046).

## Conflict of interest

The authors declare that the research was conducted in the absence of any commercial or financial relationships that could be construed as a potential conflict of interest.

## Publisher’s note

All claims expressed in this article are solely those of the authors and do not necessarily represent those of their affiliated organizations, or those of the publisher, the editors and the reviewers. Any product that may be evaluated in this article, or claim that may be made by its manufacturer, is not guaranteed or endorsed by the publisher.
