# Supplementary material for: Emerging roles of alternative RNA splicing in oral squamous cell carcinoma
Source: Front Oncol. 2022 Nov 25;12:1019750. doi: 10.3389/fonc.2022.1019750 (PMC9732560; doi:10.3389/fonc.2022.1019750)
Supplement: Supplementary file 1 [file Table_1.docx]

Table S1. The splicing factors involved in OSCC and the associated clinical impacts.

| Gene | Expression (OSCC vs. normal) | Function | Clinical impact | Reference |
| --- | --- | --- | --- | --- |
| *SRSF1* | Up | Promoting proliferation, invasion, and EMT | Unknown | (1) |
| *SRSF3* | Up | Promoting proliferation, migration, and EMT; Inhibiting autophagy | Positively associated with disease progression and negatively associated with prognosis | (2-4) |
| *SRSF5* | Up | Promoting proliferation | Unknown | (5) |
| *HNRNPA1* | Up | Promoting proliferation | Unknown | (6) |
| *HNRNPC* | Up | Promoting proliferation, migration, invasion, and EMT | Positively associated with disease progression and negatively associated with prognosis | (7) |
| *HNRNPD* | Up | Promoting cell growth | Positively associated with disease progression and negatively associated with prognosis | (8) |
| *HNRNPE1* | Down | Inhibiting tumor progression | Unknown | (9, 10) |
| *HNRNPG* | Down | Inhibiting proliferation | Unknown | (11) |
| *HNRNPK* | Up | Promoting proliferation. Enhancing drug-induced apoptosis | Negatively associated with prognosis | (12, 13) |
| *HNRNPL* | Up | Promoting proliferation and migration | Unknown | (2, 14) |
| *LSM12* | Up | Promoting proliferation, migration, and invasion | Unknown | (15) |
| *ESRP1* | Up in tumor,  down in invasive fronts | Suppressing cell motility | Unknown | (16) |
| *ESRP2* |  |  | Unknown |  |

Reference:

1. Zhang Y, Wang A, Zhang X, Wang X, Zhang J, Ma J. Lncrna Linc01296 Promotes Oral Squamous Cell Carcinoma Development by Binding with Srsf1. *Biomed Res Int* (2021) 2021:6661520. doi: 10.1155/2021/6661520

2. Jia R, Zhang S, Liu M, Zhang Y, Liu Y, Fan M, et al. Hnrnp L Is Important for the Expression of Oncogene Srsf3 and Oncogenic Potential of Oral Squamous Cell Carcinoma Cells. *Sci Rep* (2016) 6:35976. doi: 10.1038/srep35976

3. Peiqi L, Zhaozhong G, Yaotian Y, Jun J, Jihua G, Rong J. Expression of Srsf3 Is Correlated with Carcinogenesis and Progression of Oral Squamous Cell Carcinoma. *Int J Med Sci* (2016) 13(7):533-9. doi: 10.7150/ijms.14871

4. Guo J, Wang X, Jia J, Jia R. Underexpression of Srsf3 and Its Target Gene Rbmx Predicts Good Prognosis in Patients with Head and Neck Cancer. *J Oral Sci* (2020) 62(2):175-9. doi: 10.2334/josnusd.18-0485

5. Yang S, Jia R, Bian Z. Srsf5 Functions as a Novel Oncogenic Splicing Factor and Is Upregulated by Oncogene Srsf3 in Oral Squamous Cell Carcinoma. *Biochim Biophys Acta Mol Cell Res* (2018) 1865(9):1161-72. doi: 10.1016/j.bbamcr.2018.05.017

6. Yu C, Guo J, Liu Y, Jia J, Jia R, Fan M. Oral Squamous Cancer Cell Exploits Hnrnp A1 to Regulate Cell Cycle and Proliferation. *J Cell Physiol* (2015) 230(9):2252-61. doi: 10.1002/jcp.24956

7. Huang GZ, Wu QQ, Zheng ZN, Shao TR, Chen YC, Zeng WS, et al. M6a-Related Bioinformatics Analysis Reveals That Hnrnpc Facilitates Progression of Oscc Via Emt. *Aging* (2020) 12(12):11667-84. doi: 10.18632/aging.103333

8. Kumar M, Matta A, Masui O, Srivastava G, Kaur J, Thakar A, et al. Nuclear Heterogeneous Nuclear Ribonucleoprotein D Is Associated with Poor Prognosis and Interactome Analysis Reveals Its Novel Binding Partners in Oral Cancer. *J Transl Med* (2015) 13:285. doi: 10.1186/s12967-015-0637-3

9. Wang X, Guo J, Che X, Jia R. Pcbp1 Inhibits the Expression of Oncogenic Stat3 Isoform by Targeting Alternative Splicing of Stat3 Exon 23. *Int J Biol Sci* (2019) 15(6):1177-86. doi: 10.7150/ijbs.33103

10. Li Z, Wang X, Jia R. Poly(Rc) Binding Protein 1 Represses the Translation of Stat3 through 5' Utr. *Curr Gene Ther* (2022). doi: 10.2174/1566523222666220511162934

11. Shin KH, Kang MK, Kim RH, Christensen R, Park NH. Heterogeneous Nuclear Ribonucleoprotein G Shows Tumor Suppressive Effect against Oral Squamous Cell Carcinoma Cells. *Clinical Cancer Research* (2006) 12(10):3222-8. doi: 10.1158/1078-0432.Ccr-05-2656

12. Matta A, Tripathi SC, DeSouza LV, Grigull J, Kaur J, Chauhan SS, et al. Heterogeneous Ribonucleoprotein K Is a Marker of Oral Leukoplakia and Correlates with Poor Prognosis of Squamous Cell Carcinoma. *International Journal of Cancer* (2009) 125(6):1398-406. doi: 10.1002/ijc.24517

13. Wu CS, Chang KP, Chen LC, Chen CC, Liang Y, Hseuh C, et al. Heterogeneous Ribonucleoprotein K and Thymidine Phosphorylase Are Independent Prognostic and Therapeutic Markers for Oral Squamous Cell Carcinoma. *Oral Oncol* (2012) 48(6):516-22. doi: 10.1016/j.oraloncology.2012.01.005

14. Xu L, Shen J, Jia J, Jia R. Inclusion of Hnrnp L Alternative Exon 7 Is Associated with Good Prognosis and Inhibited by Oncogene Srsf3 in Head and Neck Squamous Cell Carcinoma. *Biomed Res Int* (2019) 2019:9612425. doi: 10.1155/2019/9612425

15. Dong Y, Xue L, Zhang Y, Liu C, Zhang Y, Jiang N, et al. Identification of Rna-Splicing Factor Lsm12 as a Novel Tumor-Associated Gene and a Potent Biomarker in Oral Squamous Cell Carcinoma (Oscc). *#N/A* (2022) 41(1):150. doi: 10.1186/s13046-022-02355-9

16. Ishii H, Saitoh M, Sakamoto K, Kondo T, Katoh R, Tanaka S, et al. Epithelial Splicing Regulatory Proteins 1 (Esrp1) and 2 (Esrp2) Suppress Cancer Cell Motility Via Different Mechanisms. *J Biol Chem* (2014) 289(40):27386-99. doi: 10.1074/jbc.M114.589432
