# Supplementary material for: Emerging roles of alternative RNA splicing in oral squamous cell carcinoma
Source: Front Oncol. 2022 Nov 25;12:1019750. doi: 10.3389/fonc.2022.1019750 (PMC9732560; doi:10.3389/fonc.2022.1019750)
Supplement: Supplementary file 2 [file Table_2.docx]

Table S2. Alternative spliced genes involved in OSCC and their functions.

| Gene | Splicing variant | Splicing events | Isoform expression level (OSCC) | Functions and clinical impacts | Reference |
| --- | --- | --- | --- | --- | --- |
| *MCL1* | Mcl-1L | Full-length | Overexpression | Anti-apoptotic and associated with poor overall survival | (1-5) |
|  | Mcl-1S | Exon 2 skipping | Low expression | Pro-apoptotic |  |
|  | Mcl-1ES | Partial exon 1 skipping | Very low expression | Pro-apoptotic |  |
| *Survivin (BIRC5)* | Survivin-wt | Full-length | High expression | Anti-apoptotic | (6-10) |
|  | Survivin-ΔEx3 | Exon 3 skipping | High expression | Anti-apoptotic |  |
|  | Survivin-2B | Partial intron 2 retention | 2B high expressed | Pro-apoptotic |  |
|  | Survivin-3B | Partial intron 3 retention | High expression | Anti-apoptotic and associated with poor differentiation and lymph node metastases |  |
|  | Survivin-2α | Exon 3 and 4 skipping, and partial intron 2 retention | High expression | Pro-apoptotic |  |
|  | Survivin-3α | Retain intronic sequence of survivin-2B and an additional 32 nucleotides from intron 2 | High expression | Unknown |  |
| *P63* | TAp63(α/β/γ) | Alternative promoter | Low expression | Tumor suppressor | (11-17) |
|  | ΔNp63(α/β/γ) | Alternative promoter | ΔNp63α is highly expressed | ΔNp63α inhibits invasion and EMT, ΔNp63β inhibits OSCC cell invasion and metastasis |  |
|  | ΔNp73L | ΔNp63 exon 4 skipping | Frequently expressed in metastatic tumors | Unknown |  |
|  | Δ4Tap63 | TAp63 exon 4 skipping | Frequently expressed in metastatic tumors | Unknown |  |
| *STAT3* | STAT3α | Full-length | High expression | Promoting cancer cell proliferation | (18, 19) |
|  | STAT3β | Alternative 3’ splice site of exon 23 | Low expression | Inhibiting cancer cell proliferation |  |
| *USO1* | USO1-FL | Full-length | High expression | Promoting cell proliferation and tumorigenesis | (20) |
|  | USO1-DE15 | Exon 15 skipping | N/A | Inhibiting cell proliferation and tumorigenesis |  |
| *ORAOV1* | ORAOV1 | Full length | High expression | Required for cell proliferation, tumor growth, and angiogenesis | (21-23) |
|  | ORAOV1-A | Exon 3 skipping | High expression | Associated with poor differentiation |  |
|  | ORAOV1-B | Exon 2 and exon 3 skipping | High expression | Promoting EMT and metastasis |  |
| *H19* | H19-L | Full-length | High expression | Promoting cancer cell proliferation, EMT, and metastasis | (24, 25) |
|  | H19-S | Exon 4 skipping | High expression | Promoting cancer cell proliferation and EMT |  |

Reference:

1. Bae J, Leo CP, Hsu SY, Hsueh AJ. Mcl-1s, a Splicing Variant of the Antiapoptotic Bcl-2 Family Member Mcl-1, Encodes a Proapoptotic Protein Possessing Only the Bh3 Domain. *J Biol Chem* (2000) 275(33):25255-61. doi: 10.1074/jbc.M909826199

2. Kim JH, Sim SH, Ha HJ, Ko JJ, Lee K, Bae J. Mcl-1es, a Novel Variant of Mcl-1, Associates with Mcl-1l and Induces Mitochondrial Cell Death. *FEBS Lett* (2009) 583(17):2758-64. doi: 10.1016/j.febslet.2009.08.006

3. Mallick S, Patil R, Gyanchandani R, Pawar S, Palve V, Kannan S, et al. Human Oral Cancers Have Altered Expression of Bcl-2 Family Members and Increased Expression of the Anti-Apoptotic Splice Variant of Mcl-1. *J Pathol* (2009) 217(3):398-407. doi: 10.1002/path.2459

4. Palve VC, Teni TR. Association of Anti-Apoptotic Mcl-1l Isoform Expression with Radioresistance of Oral Squamous Carcinoma Cells. *Radiat Oncol* (2012) 7:135. doi: 10.1186/1748-717x-7-135

5. Palve V, Mallick S, Ghaisas G, Kannan S, Teni T. Overexpression of Mcl-1l Splice Variant Is Associated with Poor Prognosis and Chemoresistance in Oral Cancers. *PLoS One* (2014) 9(11):e111927. doi: 10.1371/journal.pone.0111927

6. Mahotka C, Wenzel M, Springer E, Gabbert HE, Gerharz CD. Survivin-Deltaex3 and Survivin-2b: Two Novel Splice Variants of the Apoptosis Inhibitor Survivin with Different Antiapoptotic Properties. *Cancer Res* (1999) 59(24):6097-102.

7. Badran A, Yoshida A, Ishikawa K, Goi T, Yamaguchi A, Ueda T, et al. Identification of a Novel Splice Variant of the Human Anti-Apoptopsis Gene Survivin. *Biochem Biophys Res Commun* (2004) 314(3):902-7. doi: 10.1016/j.bbrc.2003.12.178

8. Caldas H, Honsey LE, Altura RA. Survivin 2alpha: A Novel Survivin Splice Variant Expressed in Human Malignancies. *Mol Cancer* (2005) 4(1):11. doi: 10.1186/1476-4598-4-11

9. Huang Y, Chen X, Chen N, Nie L, Xu M, Zhou Q. Expression and Prognostic Significance of Survivin Splice Variants in Diffusely Infiltrating Astrocytoma. *J Clin Pathol* (2011) 64(11):953-9. doi: 10.1136/jclinpath-2011-200066

10. Mishra R, Palve V, Kannan S, Pawar S, Teni T. High Expression of Survivin and Its Splice Variants Survivin Deltaex3 and Survivin 2 B in Oral Cancers. *Oral Surg Oral Med Oral Pathol Oral Radiol* (2015) 120(4):497-507. doi: 10.1016/j.oooo.2015.06.027

11. Higashikawa K, Yoneda S, Tobiume K, Taki M, Shigeishi H, Kamata N. Snail-Induced Down-Regulation of Deltanp63alpha Acquires Invasive Phenotype of Human Squamous Cell Carcinoma. *Cancer Res* (2007) 67(19):9207-13. doi: 10.1158/0008-5472.CAN-07-0932

12. Goto Y, Kawano S, Matsubara R, Kiyosue T, Hirano M, Jinno T, et al. Possible Involvement of Deltanp63 Downregulation in the Invasion and Metastasis of Oral Squamous Cell Carcinoma Via Induction of a Mesenchymal Phenotype. *Clin Exp Metastasis* (2014) 31(3):293-306. doi: 10.1007/s10585-013-9628-z

13. Sakamoto T, Kawano S, Matsubara R, Goto Y, Jinno T, Maruse Y, et al. Critical Roles of Wnt5a-Ror2 Signaling in Aggressiveness of Tongue Squamous Cell Carcinoma and Production of Matrix Metalloproteinase-2 Via Deltanp63beta-Mediated Epithelial-Mesenchymal Transition. *Oral Oncol* (2017) 69:15-25. doi: 10.1016/j.oraloncology.2017.03.019

14. Hashiguchi Y, Kawano S, Goto Y, Yasuda K, Kaneko N, Sakamoto T, et al. Tumor-Suppressive Roles of Deltanp63beta-Mir-205 Axis in Epithelial-Mesenchymal Transition of Oral Squamous Cell Carcinoma Via Targeting Zeb1 and Zeb2. *J Cell Physiol* (2018) 233(10):6565-77. doi: 10.1002/jcp.26267

15. Nylander K, Coates PJ, Hall PA. Characterization of the Expression Pattern of P63 Alpha and Delta Np63 Alpha in Benign and Malignant Oral Epithelial Lesions. *Int J Cancer* (2000) 87(3):368-72.

16. Romano RA, Solomon LW, Sinha S. Tp63 in Oral Development, Neoplasia, and Autoimmunity. *J Dent Res* (2012) 91(2):125-32. doi: 10.1177/0022034511411302

17. Chen YK, Hsue SS, Lin LM. Expression of P63 (Ta and Deltan Isoforms) in Human Primary Well Differentiated Buccal Carcinomas. *Int J Oral Maxillofac Surg* (2004) 33(5):493-7. doi: 10.1016/j.ijom.2003.10.023

18. Wang X, Guo J, Che X, Jia R. Pcbp1 Inhibits the Expression of Oncogenic Stat3 Isoform by Targeting Alternative Splicing of Stat3 Exon 23. *Int J Biol Sci* (2019) 15(6):1177-86. doi: 10.7150/ijbs.33103

19. Li Z, Wang X, Jia R. Poly(Rc) Binding Protein 1 Represses the Translation of Stat3 through 5' Utr. *Curr Gene Ther* (2022). doi: 10.2174/1566523222666220511162934

20. Dong Y, Xue L, Zhang Y, Liu C, Zhang Y, Jiang N, et al. Identification of Rna-Splicing Factor Lsm12 as a Novel Tumor-Associated Gene and a Potent Biomarker in Oral Squamous Cell Carcinoma (Oscc). *#N/A* (2022) 41(1):150. doi: 10.1186/s13046-022-02355-9

21. Luo X, Jiang Y, Chen F, Wei Z, Qiu Y, Xu H, et al. Oraov1-B Promotes Oscc Metastasis Via the Nf-Κb-Tnfα Loop. *J Dent Res* (2021) 100(8):858-67. doi: 10.1177/0022034521996339

22. Jiang L, Yang HS, Wang Z, Zhou Y, Zhou M, Zeng X, et al. Oraov1-a Correlates with Poor Differentiation in Oral Cancer. *J Dent Res* (2009) 88(5):433-8. doi: 10.1177/0022034509336994

23. Jiang L, Zeng X, Yang H, Wang Z, Shen J, Bai J, et al. Oral Cancer Overexpressed 1 (Oraov1): A Regulator for the Cell Growth and Tumor Angiogenesis in Oral Squamous Cell Carcinoma. *Int J Cancer* (2008) 123(8):1779-86. doi: 10.1002/ijc.23734

24. Zhou W, Wang XZ, Fang BM. A Variant of H19 Transcript Regulates Emt and Oral Cancer Progression. *Oral Dis* (2022) 28(1):116-24. doi: 10.1111/odi.13739

25. Zhang DM, Lin ZY, Yang ZH, Wang YY, Wan D, Zhong JL, et al. Incrna H19 Promotes Tongue Squamous Cell Carcinoma Progression through Beta-Catenin/Gsk3beta/Emt Signaling Via Association with Ezh2. *Am J Transl Res* (2017) 9(7):3474-86.
