# Supplementary material for: Emerging roles of alternative RNA splicing in oral squamous cell carcinoma
Source: Front Oncol. 2022 Nov 25;12:1019750. doi: 10.3389/fonc.2022.1019750 (PMC9732560; doi:10.3389/fonc.2022.1019750)
Supplement: Supplementary file 3 [file Table_3.docx]

Table S3. Splice modulators used in different types of tumor cells.

| Target | Category | Compound name | Tumor and cell type | Reference |
| --- | --- | --- | --- | --- |
| SF3B1 | Small molecules | Spliceostatin A | Chronic lymphocytic leukaemia | (1) |
| SF3B1 | Small molecules | Meayamycin B | Non-small cell lung cancer and head and neck cancer cells | (2, 3) |
| SF3B1 | Small molecules | Pladienolide-B | Cervical carcinoma cells and lymphocytic leukaemia cells | (4, 5) |
| SF3B1 | Small molecules | GEX1A | HeLa cells | (6) |
| SF3B1 | Small molecules | H3B-8800 | Pancreatic cancer cells and acute myeloid leukemia cells | (7) |
| SF3B1 | Small molecules | Sudemycins | Pediatric rhabdomyosarcoma cells | (8) |
| SF3B1 | Small molecules | FD-895 | Lymphocytic leukaemia cells | (5) |
| RBM39 | Small molecules | Indisulam, tasisulam, E7820, and chloroquinoxaline sulfonamide | Colorectal cancer cell line, leukemia cells | (9, 10) |
| SRSF3 | Antisense oligonucleotides | -- | Oral squamous cell carcinoma | (11, 12) |
| SRSF3 | Small molecules | SFI003 | Colorectal cancer | (13) |
| STAT3 | Antisense oligonucleotides | -- | Breast cancer | (14) |
| BCL-X | Antisense oligonucleotides | -- | Mouse melanoma cells | (15) |

Reference:

1. Larrayoz M, Blakemore SJ, Dobson RC, Blunt MD, Rose-Zerilli MJ, Walewska R, et al. The Sf3b1 Inhibitor Spliceostatin a (Ssa) Elicits Apoptosis in Chronic Lymphocytic Leukaemia Cells through Downregulation of Mcl-1. *Leukemia* (2016) 30(2):351-60. doi: 10.1038/leu.2015.286

2. Gao Y, Koide K. Chemical Perturbation of Mcl-1 Pre-Mrna Splicing to Induce Apoptosis in Cancer Cells. *ACS Chem Biol* (2013) 8(5):895-900. doi: 10.1021/cb300602j

3. Gao Y, Trivedi S, Ferris RL, Koide K. Regulation of Hpv16 E6 and Mcl1 by Sf3b1 Inhibitor in Head and Neck Cancer Cells. *Sci Rep* (2014) 4:6098. doi: 10.1038/srep06098

4. Zhang Q, Di C, Yan J, Wang F, Qu T, Wang Y, et al. Inhibition of Sf3b1 by Pladienolide B Evokes Cycle Arrest, Apoptosis Induction and P73 Splicing in Human Cervical Carcinoma Cells. *Artif Cells Nanomed Biotechnol* (2019) 47(1):1273-80. doi: 10.1080/21691401.2019.1596922

5. Kashyap MK, Kumar D, Villa R, La Clair JJ, Benner C, Sasik R, et al. Targeting the Spliceosome in Chronic Lymphocytic Leukemia with the Macrolides Fd-895 and Pladienolide-B. *Haematologica* (2015) 100(7):945-54. doi: 10.3324/haematol.2014.122069

6. Hasegawa M, Miura T, Kuzuya K, Inoue A, Won Ki S, Horinouchi S, et al. Identification of Sap155 as the Target of Gex1a (Herboxidiene), an Antitumor Natural Product. *ACS Chemical Biology* (2011) 6(3):229-33. doi: 10.1021/cb100248e

7. Seiler M, Yoshimi A, Darman R, Chan B, Keaney G, Thomas M, et al. H3b-8800, an Orally Available Small-Molecule Splicing Modulator, Induces Lethality in Spliceosome-Mutant Cancers. *Nat Med* (2018) 24(4):497-504. doi: 10.1038/nm.4493

8. Fan L, Lagisetti C, Edwards CC, Webb TR, Potter PM. Sudemycins, Novel Small Molecule Analogues of Fr901464, Induce Alternative Gene Splicing. *ACS Chem Biol* (2011) 6(6):582-9. doi: 10.1021/cb100356k

9. Han T, Goralski M, Gaskill N, Capota E, Kim J, Ting TC, et al. Anticancer Sulfonamides Target Splicing by Inducing Rbm39 Degradation Via Recruitment to Dcaf15. *Science* (2017) 356(6336). doi: 10.1126/science.aal3755

10. Uehara T, Minoshima Y, Sagane K, Sugi NH, Mitsuhashi KO, Yamamoto N, et al. Selective Degradation of Splicing Factor Caper Alpha by Anticancer Sulfonamides. *Nat Chem Biol* (2017) 13(6):675-+. doi: 10.1038/nchembio.2363

11. Guo J, Che X, Wang X, Jia R. Inhibition of the Expression of Oncogene Srsf3 by Blocking an Exonic Splicing Suppressor with Antisense Oligonucleotides. *RSC Adv* (2018) 8(13):7159-63. doi: 10.1039/c7ra11267j

12. Sun Y, Yan L, Guo J, Shao J, Jia R. Downregulation of Srsf3 by Antisense Oligonucleotides Sensitizes Oral Squamous Cell Carcinoma and Breast Cancer Cells to Paclitaxel Treatment. *Cancer Chemother Pharmacol* (2019) 84(5):1133-43. doi: 10.1007/s00280-019-03945-9

13. Zhang Y, Wang M, Meng F, Yang M, Chen Y, Guo X, et al. A Novel Srsf3 Inhibitor, Sfi003, Exerts Anticancer Activity against Colorectal Cancer by Modulating the Srsf3/Dhcr24/Ros Axis. *Cell Death Discov* (2022) 8(1):238. doi: 10.1038/s41420-022-01039-9

14. Zammarchi F, de Stanchina E, Bournazou E, Supakorndej T, Martires K, Riedel E, et al. Antitumorigenic Potential of Stat3 Alternative Splicing Modulation. *Proc Natl Acad Sci U S A* (2011) 108(43):17779-84. doi: 10.1073/pnas.1108482108

15. Bauman JA, Li SD, Yang A, Huang L, Kole R. Anti-Tumor Activity of Splice-Switching Oligonucleotides. *Nucleic Acids Res* (2010) 38(22):8348-56. doi: 10.1093/nar/gkq731
